# Supplementary figures and images for: Hemodynamic Adaptations Induced by Short-Term Run Interval Training in College Students
Source: Int J Environ Res Public Health. 2020 Jun 27;17(13):4636. doi: 10.3390/ijerph17134636 (PMC7369875; doi:10.3390/ijerph17134636)

A)

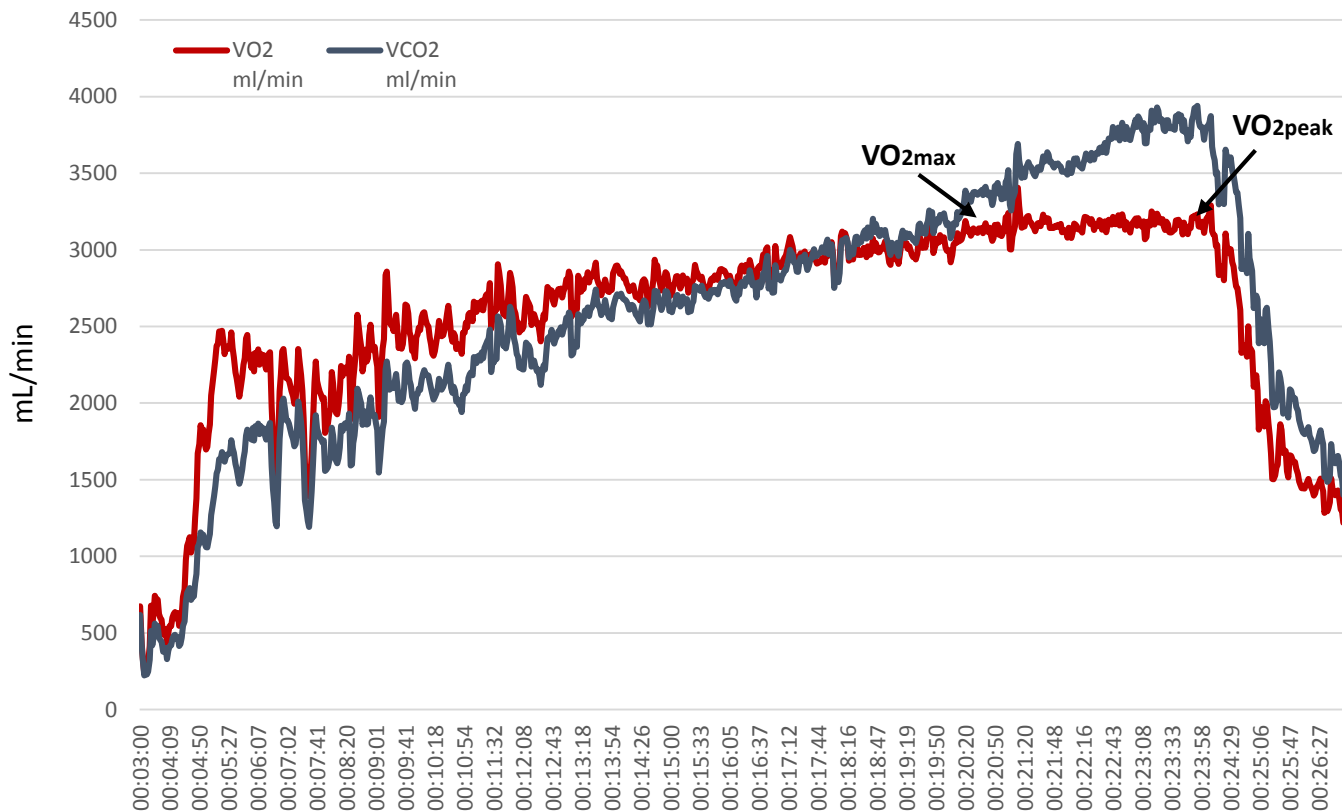

B)

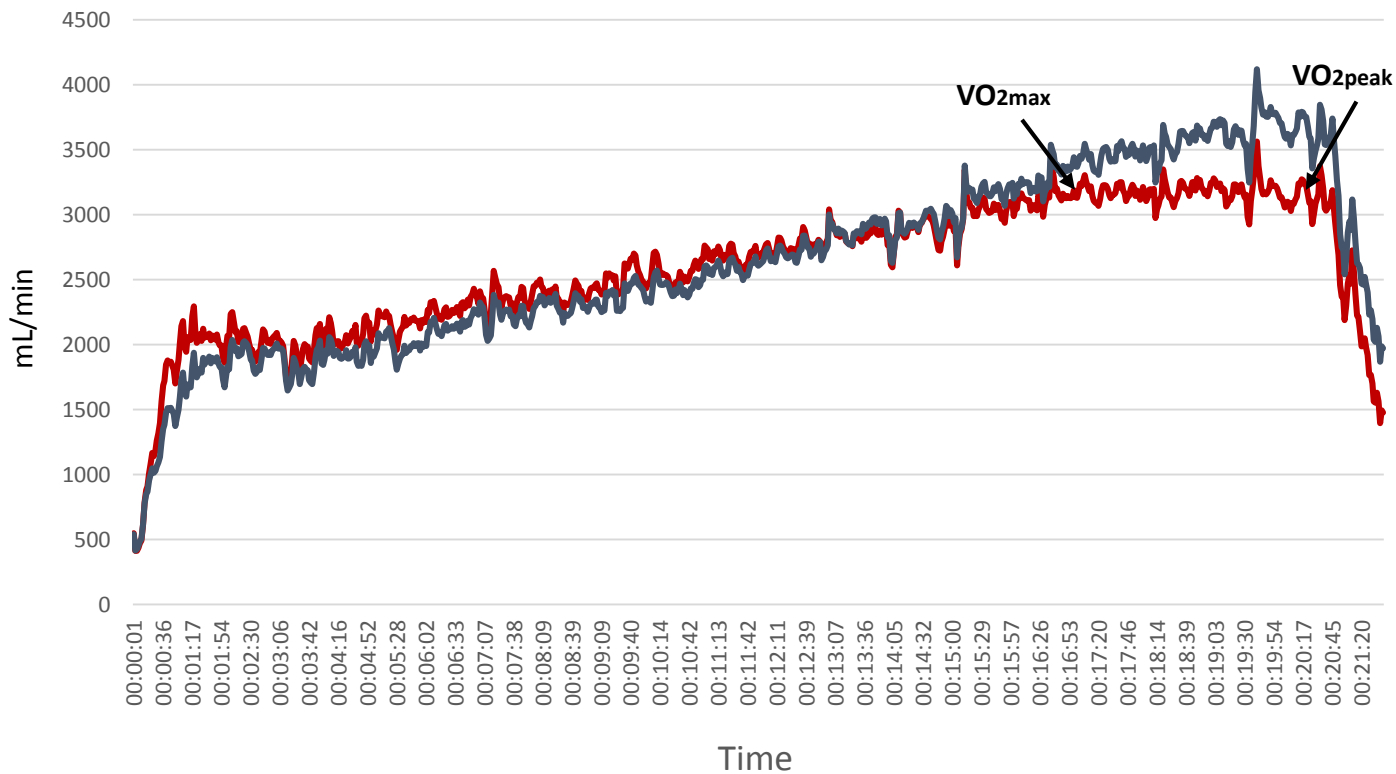

Supplement: Supplementary file 1 [file ijerph-17-04636-s001.pdf]
